# 中国肺癌脑转移诊治专家共识（2017年版）

**DOI:** 10.3779/j.issn.1009-3419.2017.01.01

**Published:** 2017-01-20

**Authors:** 远凯 石, 燕 孙, 金明 于, 翠敏 丁, 智勇 马, 子平 王, 东 王, 征 王, 孟昭 王, 燕 王, 铀 卢, 斌 艾, 继锋 冯, 云鹏 刘, 晓晴 刘, 基巍 刘, 钢 伍, 宝林 曲, 学记 李, 恩孝 李, 薇 李, 勇 宋, 公琰 陈, 正堂 陈, 骏 陈, 萍 余, 宁 吴, 密璐 吴, 文华 肖, 建平 肖, 力 张, 阳 张, 沂平 张, 树才 张, 霞 宋, 荣城 罗, 彩存 周, 宗玫 周, 琼 赵, 成平 胡, 毅 胡, 立功 聂, 其森 郭, 建华 常, 诚 黄, 宝惠 韩, 晓红 韩, 功 黎, 昱 黄, 幼梧 史

**Affiliations:** 1 100021 北京，国家癌症中心/中国医学科学院北京协和医学院肿瘤医院，抗肿瘤分子靶向药物临床研究北京市重点实验室 National Cancer Center/Cancer Hospital, Chinese Academy of Medical Sciences & Peking Union Medical College, Beijing Key Laboratory of Clinical Study on Anticancer Molecular Targeted Drugs, 100021 Beijing, China; 2 250117 济南，山东省肿瘤医院 Shandong Province Cancer Hospital, 250117 Jinan, China; 3 050000 石家庄，河北医科大学第四医院 The Fourth Hospital of Hebei Medical University, 050000 Shijiazhuang, China; 4 450008 郑州，河南省肿瘤医院 Henan Province Cancer Hospital, 450008 Zhengzhou, China; 5 100142 北京，北京大学肿瘤医院 Beijing Cancer Hospital, 100142 Beijing, China; 6 400042 重庆，第三军医大学大坪医院 Daping Hospital, Third Military Medical University, 400042 Chongqing, China; 7 100730 北京，国家老年医学中心/北京医院 National Center for Geriatric Medicine/Beijing Hospital, 100730 Beijing, China; 8 100730 北京，北京协和医院 Peking Union Medical College Hospital, 100730 Beijing, China; 9 610041 成都，四川大学华西医院 West China Hospital of Sichuan University, 610041 Chengdu, China; 10 210009 南京，江苏省肿瘤医院 Jiangsu Cancer Hospital, 210009 Nanjing, China; 11 110001 沈阳，中国医科大学附属第一医院 The First Hospital of China Medical University, 110001 Shenyang, China; 12 100071 北京，中国人民解放军第307医院 The 307th Hospital of Chinese People's Liberation Army, 100071 Beijing, China; 13 116011 大连，大连医科大学附属第一医院 The First Affiliated Hospital of Dalian Medical University, 116011 Dalian, China; 14 430022 武汉，华中科技大学协和医院 Huazhong University of Science and Technology Union Hospital, 430022 Wuhan, China; 15 100853 北京，中国人民解放军总医院 Chinese People's Liberation Army General Hospital, 100853 Beijing, China; 16 100021 北京，国家癌症中心/中国医学科学院北京协和医学院肿瘤医院 National Cancer Center/Cancer Hospital, Chinese Academy of Medical Sciences & Peking Union Medical College, 100021 Beijing, China; 17 710061 西安，西安交通大学第一附属医院 The First Affiliated Hospital of Xi ' an Jiaotong University, 710061 Xi'an, China; 18 130021 长春，吉林大学第一医院 The First Hospital of Jilin University, 130021 Changchun, China; 19 210002 南京，中国人民解放军南京总医院 Nanjing General Hospital, 210002 Nanjing, China; 20 150081 哈尔滨，哈尔滨医科大学附属肿瘤医院 Harbin Medical University Cancer Hospital, 150081 Harbin, China; 21 400037 重庆，第三军医大学新桥医院 Xinqiao Hospital of Third Military medical University, 400037 Chongqing, China; 22 116027 大连，大连医科大学附属第二医院 The Second Hospital of Dalian Medical University, 116027 Dalian, China; 23 610047 成都，四川省肿瘤医院 Sichuan Cancer Hospital, 610047 Chengdu, China; 24 810000 西宁，青海大学附属肿瘤医院 Qinghai University Affiliated Cancer Hospital, 810000 Xining, China; 25 100048 北京，中国人民解放军总医院第一附属医院 The First Affiliated Hospital of Chinese People's Liberation Army General Hospital, 100048 Beijing, China; 26 310022 杭州，浙江省肿瘤医院 Zhejiang Cancer Hospital, 310022 Hangzhou, China; 27 101149 北京，首都医科大学附属北京胸科医院 Beijing Chest Hospital, Capital Medical University, 101149 Beijing, China; 28 030013 太原，山西省肿瘤医院 Shanxi Province Cancer Hospital, 030013 Taiyuan, China; 29 510315 广州，南方医科大学肿瘤中心 TCM-Integrated Cancer Center of Southern Medical University, 510315 Guangzhou, China; 30 200433 上海，同济大学附属上海市肺科医院 Tongji University Affiliated Shanghai Pulmonary Hospital, 200433 Shanghai, China; 31 310003 杭州，浙江大学附属第一医院 The First Affiliated Hospital, Zhejiang University, 310003 Hangzhou, China; 32 410008 长沙，中南大学湘雅医院 Xiangya Hospital Central South University, 410008 Changsha, China; 33 100034 北京，北京大学第一医院 Peking University First Hospital, 100034 Beijing, China; 34 200032 上海，复旦大学附属肿瘤医院 Fudan Universitay Shanghai Cancer Center, 200032 Shanghai, China; 35 350014 福州，福建省肿瘤医院 Fujian Cancer Hospital, 350014 Fuzhou, China; 36 200030 上海，上海交通大学附属胸科医院 Shanghai Chest Hospital, Shanghai Jiaotong University, 200030 Shanghai, China; 37 100039 北京，中国武警总医院 General Hospital of Armed Police, 100039 Beijing, China

## 一 概述

原发性肺癌（以下简称肺癌）是我国最常见的恶性肿瘤之一，肺癌最常见的远处转移部位之一是脑部。肺癌脑转移患者预后差，自然平均生存时间仅1个月-2个月。放射治疗技术的进步和分子靶向治疗等新疗法的迅速发展，为晚期肺癌脑转移提供了更多的治疗手段和更多的期待，手术、放疗及化疗等治疗手段的综合应用在一定程度上延长了肺癌脑转移患者的生存期、显著地改善了生活质量。肺癌脑转移的治疗已经成为临床关注的热点之一。《原发性肺癌诊疗规范（2011年版）》^[1]^、《中国原发性肺癌诊疗规范（2015年版）》^[2]^和《中国晚期原发性肺癌诊治专家共识（2016年版）》^[3]^的颁布，对我国原发性肺癌的规范化诊治起到了积极的促进作用。为了进一步提高我国肺癌脑转移的诊疗水平，改善肺癌脑转移患者的预后，中国医师协会肿瘤医师分会和中国抗癌协会肿瘤临床化疗专业委员会组织全国专家，制定了《中国肺癌脑转移诊治专家共识（2017年版）》。

## 二 流行病学

脑转移性肿瘤包括脑实质转移（brain metastasis, BM）和脑膜转移（leptomeningeal metastasis, LM）。脑实质转移瘤最常见的发生部位为大脑半球，其次为小脑和脑干^[4]^。脑膜转移较脑实质转移少见，但预后更差。近年来，随着肺癌发病率上升，诊疗技术不断发展，使患者生存期延长，肺癌脑转移的发生和诊断率也逐年升高。肺癌脑转移发生率明显高于黑色素瘤、乳腺癌、肾癌及结直肠癌^[5]^，20%-65%的肺癌患者在病程中会发生脑转移，是脑转移性肿瘤中最常见的类型^[6-8]^。各组织学类型肺癌脑转移的发生率存在差异，美国医疗保险监督、流行病学和最终结果（Surveillance, Epidemiology, and End Results, SEER）数据库的一项长期随访结果显示，在非转移性非小细胞肺癌（non-small cell lung cancer, NSCLC）中，肺腺癌、鳞癌及大细胞癌发生脑转移的风险分别为11%、6%及12%^[9]^。小细胞肺癌（small cell lung cancer, SCLC）首次就诊时脑转移发生率为10%，诊疗过程中为40%-50%，存活2年以上的患者脑转移达60%-80%，是影响SCLC患者生存及生活质量的重要因素之一^[10]^。

## 三 临床表现

肺癌脑实质内转移和脑膜转移临床表现有其共性又各有特点。

### 一 脑实质转移

脑实质转移瘤的临床表现主要包括共性的颅内压增高、特异性的局灶性症状和体征。

#### 1 颅内压增高

颅内压增高的症状和体征主要表现为头痛、呕吐和视神经乳头水肿。除这三个主征外，还可出现复视、黑朦、视力减退，头晕、淡漠、意识障碍，二便失禁、脉搏徐缓和血压增高等征象。症状常常呈进行性加重，当转移瘤囊性变或瘤内卒中时可出现急性颅内压增高症状。

#### 2 局灶性症状和休征

大脑半球功能区附近的转移瘤早期可出现局部刺激症状，晚期则出现神经功能破坏性症状，且不同部位肿瘤可产生不同的定位症状和体征，包括：①精神症状：常见于额叶肿瘤，可表现为性情改变、反应迟钝、痴呆等；②癫痫发作：额叶肿瘤较多见，其次为颞叶、顶叶肿瘤。可为全身阵挛性大发作或局限性发作；③感觉障碍：为顶叶转移瘤的常见症状，表现为两点辨别觉、实体觉及对侧肢体的位置觉障碍；④运动障碍：表现为肿瘤对侧肢体或肌力减弱或完全性上运动神经元瘫痪；⑤失语症：见于优势大脑半球语言中枢区转移瘤，可表现为运动性失语、感觉性失语、混合性失语和命名性失语等；⑥视野损害：枕叶及顶叶、颞叶深部肿瘤因累及视辐射，而引起对侧同象限性视野缺损或对侧同向性偏盲。

丘脑转移瘤可产生丘脑综合征，主要表现为：对侧的感觉缺失和/或刺激症状，对侧不自主运动，并可有情感与记忆障碍。

小脑转移瘤的临床表现：①小脑半球肿瘤：可出现爆破性语言、眼球震颤、患侧肢体协调动作障碍、同侧肌张力减低、腱反射迟钝、易向患侧倾倒等；②小脑蚓部肿瘤：主要表现为步态不稳、行走困难、站立时向后倾倒；③肿瘤阻塞第四脑室的早期即出现脑积水及颅内压增高表现。

脑干转移瘤大都出现交叉性瘫痪，即病灶侧脑神经周围性瘫痪和对侧肢体中枢性瘫痪及感觉障碍。根据受损脑神经可定位转移瘤的位置：如第Ⅲ对脑神经麻痹则肿瘤位于中脑；第Ⅴ、Ⅵ、Ⅶ、Ⅷ对脑神经麻痹则肿瘤位居脑桥；第Ⅸ、Ⅹ、Ⅺ、Ⅻ对脑神经麻痹则肿瘤侵犯延髓。

### 二 脑膜转移

脑膜转移患者的临床表现常因肿瘤细胞侵犯部位不同而复杂多样，缺乏特异性，有时很难与脑实质转移引起的症状和治疗原发肿瘤出现的毒副反应相鉴别；部分患者因颈肩部疼痛进行性加重而被确诊为脑膜转移。

脑膜转移的主要临床表现有：①脑实质受累及脑膜刺激表现：头痛、呕吐、颈项强直、脑膜刺激征、精神状态改变、意识朦胧、认知障碍、癫痫发作和肢体活动障碍等；②颅神经受累表现：常见的受累脑神经有视神经、动眼神经、滑车神经、外展神经、面神经、听神经等，表现为视力下降、复视、面部麻木、味觉和听觉异常、吞咽和发音困难等；③颅内压增高表现（头痛、呕吐、视乳头水肿）和脑积水压迫脑组织引起的进行性脑功能障碍表现（智力障碍、步行障碍、尿失禁）等；④如同时伴有脊膜播散则还可出现脊髓和脊神经根刺激表现，这些也有助于脑膜转移的诊断，如神经根性疼痛、节段性感觉缺损、肢体麻木、感觉性共济失调、腱反射减弱或消失、括约肌功能障碍等。

## 四 辅助检查

1.头颅磁共振成像（magnetic resonance imaging, MRI）：头颅MRI平扫典型脑转移瘤可见T1中低、T2中高异常信号，病灶周围水肿，增强扫描后可见较明显强化。增强MRI对微小病灶、水肿和脑膜转移较增强CT敏感，在肺癌脑转移的诊断、疗效评价及随访中均具有重要作用，应作为首选的影像学检查方法。

2.头颅计算机断层扫描（computed tomography, CT）：CT平扫时脑转移瘤多表现为等密度或低密度，少数为高密度灶；典型脑转移瘤在增强CT上强化明显，周围可见水肿。CT对于肺癌脑转移的诊断、疗效评价及治疗后随访具有重要意义，有头颅MRI检查禁忌症的患者应行CT检查。

3.正电子发射计算机断层扫描（positron emission tomography/CT, PET-CT）：PET-CT能够评价肿瘤及正常组织的代谢差异，有助于肿瘤的定性诊断，同时可寻找原发肿瘤。由于正常脑组织对^18^F-脱氧葡萄糖（^18^F-fluorodeoxyglucose, ^18^F-FDG，简称为FDG）呈高摄取，故FDG PET-CT对脑转移瘤、尤其是小的脑转移灶不敏感，应结合头颅MRI或增强CT扫描增加检出率。

4.腰椎穿刺及脑脊液检查：腰椎穿刺可行脑脊液压力检测，收集脑脊液并完善脑脊液常规、生化及细胞学病理诊断检查，脑转移尤其是软脑膜转移的患者可出现脑脊液压力增高、蛋白含量增高，如细胞学检查见癌细胞可明确诊断。

5.血清肿瘤标志物：肺癌相关的血清肿瘤标志物包括癌胚抗原（carcinoembryonic antigen, CEA）、细胞角蛋白片段19（cytokeratin fragment, CYFRA21-1）、鳞状上皮细胞癌抗原（squamous cell carcinoma antigen, SCC）等，SCLC具有神经内分泌特征，可有促胃泌素释放肽前体（progastrin-releasing peptide, ProGRP）、神经元特异性烯醇化酶（neuron-specific enolase, NSE）、肌酸激酶BB（creatine kinaseBB, CK-BB）以及嗜铬蛋白A（chromograninA, CgA）等的释放异常。上述肺癌相关的血清肿瘤标志物可作为监测疗效和病情变化的辅助指标。

6.分子病理检测：对于晚期腺癌或含腺癌成分的其他类型肺癌，应在诊断的同时常规进行表皮生长因子受体（epidermal growth factor receptor, *EGFR*）基因突变和间变性淋巴瘤激酶（anaplastic lymphoma kinase, *ALK*）融合基因等的检测。脑脊液标本经细胞学病理诊断后，如查见癌细胞，可以应用脑脊液标本中癌细胞和/或无细胞脑脊液上清作为基因检测的标本。

## 五 治疗

### 一 治疗原则

肺癌脑转移患者的治疗应该在全身治疗的基础上，进行针对脑转移的治疗，包括手术、全脑放疗（whole brain radiotherapy, WBRT）、立体定向放射治疗（stereotactic radiotherapy, SRT）、化疗和分子靶向治疗在内的多学科综合治疗，其目的是治疗转移病灶、改善患者症状、提高生活质量，最大程度地延长患者生存时间。

#### 1 NSCLC脑转移的治疗

对于无症状脑转移患者，可先行全身治疗：①*EGFR*基因敏感突变并且不存在耐药基因突变的晚期NSCLC患者推荐表皮生长因子受体酪氨酸激酶抑制剂（epidermal growth factor receptor tyrosine kinase inhibitors, EGFR-TKIs）一线治疗，*ALK*融合基因阳性患者推荐克唑替尼一线治疗；②*EGFR*基因敏感突变阴性、*ALK*融合基因阴性及这两个基因表达状况未知并伴有脑转移的晚期NSCLC患者，应行全身化疗。

对于有症状脑转移而颅外病灶稳定的患者，应积极行局部治疗。如脑转移瘤数目不超过3个，可采用以下治疗方案：①手术切除脑转移瘤（详见“手术治疗”）；②SRT；③SRT联合WBRT。如脑转移瘤数目多于3个，可行WBRT或SRT。

#### 2 SCLC脑转移的治疗

对于初治无症状的SCLC脑转移患者，可先行全身化疗后再行WBRT；对于有症状的SCLC脑转移患者，应积极行WBRT。之前接受过WBRT的复发患者再次进行WBRT要谨慎评估。

### 二 手术治疗

较化疗、放疗等其他治疗方法，手术具有如下优点：①全部切除转移瘤可以迅速缓解颅内高压症状，消除转移灶对周围脑组织的刺激；②获得肿瘤组织，从而明确病理诊断；③手术能通过切除全部肿瘤而达到局部治愈。

#### 1 手术适应证

（1）活检术：明确病理、分子或基因类型，指导下一步治疗。

1) 肺原发灶隐匿或虽原发灶明确但取材困难；

2) 肺原发灶病理明确，但脑部病变不典型或难于鉴别；

3) 明确是肿瘤坏死抑或复发，评估前期放、化疗效果。

（2）手术切除：脑转移瘤患者是否适合手术切除需考虑肿瘤个数、大小和部位、组织学类型、患者的全身状况等，以上因素要单独考量，但手术选择还应整合所有因素、综合权衡。值得注意的是，脑转移的患者都是晚期，手术选择应该谨慎。

1) 脑内单发、部位适合、易于切除，且肿瘤或其水肿占位效应重或导致脑积水的患者适合手术切除。而虽为单发但对放、化疗敏感的病理类型，如SCLC等可不首选手术，但下列情况除外：转移瘤和/或水肿体积大、颅内压失代偿、肿瘤卒中等濒临脑疝、危及生命者应急诊手术，为下一步放、化疗争取时间和空间。

2) 多发脑转移瘤手术治疗目前尚有争议，但一般认为：若肿瘤数目不超过3个，且手术能完全切除，则与单发脑转移瘤患者一样也能获得满意的效果。3个以上脑转移病灶治疗应首选WBRT或SRT，但如果出现肿瘤卒中、梗阻性脑积水等危及生命时，也应行手术减压。

3) 肿瘤大小：肿瘤最大径大于3 cm者，一般不适合放射治疗，宜首选手术；肿瘤最大径小于5 mm，尤其位于脑深部（丘脑、脑干等）宜首选放疗或化疗；如肿瘤最大径介于1 cm-3 cm，则根据全身状况、手术风险等综合评估来决定首选手术还是其他治疗。

4) 肿瘤部位：尽管目前借助神经导航、术中功能定位等技术，神经外科医生可以到达颅内任何一个部位，但脑深部或功能区转移瘤手术的致残率总体上仍较浅表或非功能区的手术致残率为高。因此，对位于脑干、丘脑、基底节的脑转移瘤原则上不首选手术。

#### 2 手术方法

（1）手术辅助技术

目前，多模态神经影像技术、神经导航、术中超声以及术中电生理监测等辅助措施能最大限度地减少手术副损伤，对功能区转移瘤手术十分重要。

（2）手术入路

1) 大脑皮质下转移瘤：经皮质入路，环形切开肿瘤表面薄层脑组织，全切肿瘤。但如肿瘤位居功能区，则严禁此做法，应在肿瘤表面皮质或脑沟做纵向切口，先瘤内分块切除，再全切肿瘤，尽量减少对瘤周脑组织的损伤。

2) 位于脑沟两侧或脑沟深部的转移瘤：经脑沟入路，分开脑沟，在其侧面或底部切除肿瘤。

3) 脑白质深部转移瘤，可经皮质或经脑沟入路切除。

4) 岛叶转移瘤则分开侧裂切除肿瘤。

5) 中线部位转移瘤最好经纵裂入路切除。

6) 脑室肿瘤则可经胼胝体或皮层入路切除。

7) 小脑转移瘤切除则以最短的经小脑实质径路为佳。

（3）对于脑膜转移的患者，可植入Ommaya储液囊行脑室内化疗，对合并交通性脑积水的患者可行脑室-腹腔分流术以降低颅内压、缓解症状，但脑室-腹腔分流术可能增加肿瘤腹腔转移的机会。

（4）复发脑转移瘤的再次手术

脑转移瘤的术后复发有两种情况：手术残留、肿瘤在原位复发和原发部位以外的新发脑转移瘤，如经肿瘤个数、全身状况等因素整合考量适合手术，则再次手术也能够改善患者的生活质量和预后。

### 三 放射治疗

#### 1 WBRT

WBRT是脑转移瘤的主要局部治疗措施之一，可以缓解晚期肺癌脑转移患者的神经系统症状，改善肿瘤局部控制情况。WBRT对颅内亚临床病灶有一定的控制作用，但因其受正常脑组织的剂量限制，难以根治颅内病变，约1/3脑转移患者WBRT后颅内病变未控，50%脑转移患者死于颅内病变进展。WBRT仅可延迟0.5年-1年颅内新发灶的出现，甚至有的患者在WBRT过程中又出现新的颅内转移灶^[11]^。在SRT及各种分子靶向治疗等综合手段迅速发展的今天，许多NSCLC脑转移患者生存期明显延长，脑转移进展时间延迟，即使对于多发性脑转移瘤的患者，约50%亦可避免接受WBRT^[12]^。故对于就医条件许可、随诊方便的NSCLC脑转移患者，应尽可能推迟WBRT，留待作为挽救治疗手段。WBRT的适应证包括：①NSCLC脑转移患者立体定向放射外科治疗（stereotactic radiosurgery, SRS）失败后的挽救治疗；②多于3个病灶的NSCLC脑转移患者的初始治疗，联合SRS局部加量；③NSCLC脑转移患者颅内转移灶切除术后的辅助治疗；④对广泛脑膜转移的肺癌患者综合应用WBRT与椎管内化疗，对有脊膜转移的肺癌患者可行全脑全脊髓放疗；⑤广泛期SCLC伴有脑转移的患者，无论是否有症状，也无论转移病灶多少，均可行WBRT，SCLC发生脑转移时WBRT通常是首选治疗手段，主要原因是多发脑转移的发生概率高；⑥SCLC患者之前接受过脑预防照射（prophylactic cranial irradiation, PCI）者，之后出现多发脑转移时，可慎重再次选择WBRT。

关于肺癌脑转移患者WBRT照射剂量及分割方式，目前临床上总体共识为30 Gy/10 f和40 Gy/20 f可作为大部分患者的方案，美国国立综合癌症网络（National Comprehensive Cancer Network, NCCN）指南中加入37.5 Gy/15 f的分割方式。对预后差的脑转移患者如多发、老年患者可考虑予以20 Gy/5 f的短疗程WBRT分割方案。然而，对于初诊肺癌脑转移且未行全身治疗的患者，不建议予以短疗程WBRT，主要考虑该原发肿瘤可能对全身治疗比较敏感，患者可能有一定的生存期，短疗程放疗会给患者带来晚期毒性反应^[13]^。全脑全脊髓放疗的剂量和分割方式为全脑40 Gy/2 Gy/20 f、全脊髓36 Gy/1.8 Gy/20 f。治疗中应充分考虑患者的症状、脑转移病灶的数目、脑水肿情况及对认知功能的影响，合理地选择剂量分割，并结合术后、SRT进行进一步的研究。

随着肺癌脑转移患者的生存时间逐渐延长，必须注意到WBRT导致的神经认知功能损伤，主要表现为短期及晚期记忆力下降，降低患者的生活质量，这可能与照射诱导海马结构损伤有关^[14]^。因此，多项研究探索保护海马的WBRT，将海马区最大剂量限制在9 Gy-16 Gy，可降低神经认知功能下降的发生率，且治疗后海马区出现转移的概率仅为1.4%-4.5%^[15-18]^。

#### 2 SRT

SRT在脑转移的治疗包括：SRS、分次立体定向放射治疗（fractionated stereotactic radiotherapy, FSRT）和大分割立体定向放射治疗（hypofractionated stereotactic radiotherapy, HSRT）。2006年美国放射肿瘤学会（American Society for Radiation Oncology, ASTRO）和美国神经外科医师协会（American Association of Neurological Surgeons, AANS）联合定义SRS为单次剂量或者2-5分次的SRT。SRS具有定位精确、剂量集中、损伤相对较小等优点，能够很好地保护周围正常组织，控制局部肿瘤进展，缓解神经系统症状，且对神经认知功能影响小，已逐渐成为脑转移瘤的重要治疗手段。最初SRS仅推荐用于单发小体积转移瘤的治疗，而随着放疗机器及图像引导设备的日渐先进，SRS与FSRT的适应证越来越广泛。目前SRT/FSRT治疗的主要适应证为：①单发直径4 cm-5 cm以下的转移瘤（SCLC除外）的初程治疗；②≤4个转移灶的初程治疗；③WBRT失败后的挽救治疗；④颅内转移灶切除术后的辅助治疗；⑤既往接受SRS治疗的患者疗效持续时间超过6个月，且影像学认为肿瘤复发而不是坏死，可再次考虑SRS；⑥局限的脑膜转移灶WBRT基础上的局部加量治疗。

对于1个-4个病灶的脑转移瘤，单纯SRT比单纯WBRT具有生存优势，且能更好地保留认知功能^[19-22]^。多项研究表明，5个以上甚至10个以上的转移病灶应用SRT作为初程治疗亦可达到不劣于寡转移灶的局部控制率（disease control rate, DCR）^[23-26]^。因此，SRT在多发脑转移瘤的治疗中展现了越来越大的潜力。不可否认的是，接受单纯SRT治疗的患者颅内远处失败率高于WBRT，因此，对于多发性脑转移瘤患者，初程SRT后需进行密切随访，一般2个月-3个月复查一次，监测颅内新发病灶的发生，并且应对患者进行颅内远转风险分层。国内外研究提出的高危因素有：大于4个转移灶、颅外疾病未控、转移灶体积大于6 cm3以及原发灶诊断和脑转移诊断时间小于60个月等^[27-29]^，推荐对于高危患者行SRT联合WBRT，反之则行单纯SRT。

对于大体积病灶（通常为＞3 cm），单次的SRS难以达到良好的局部控制，且治疗毒性明显提高，因此建议采用FSRT。目前文献报道采用SRS/FSRT/HSRT治疗大体积脑转移瘤的1年DCR为61%-96.6%，不良反应可耐受^[30-34]^。FSRT的单次剂量建议3.5 Gy-4 Gy，总剂量52.5 Gy-60 Gy。对于体积巨大的病灶，可采用分段放疗的模式，给予40 Gy-50 Gy剂量后休息1个月-2个月，待肿瘤缩小后再进行补量。

由于颅内肿瘤具有难以完整切除的特性，单纯手术治疗后患者极易复发，故术后行术区局部调强适形放疗（对术区较大者）或FSRT治疗实为必要，尤其对于一般状况良好和颅外疾病控制的预后较好的患者。对于孤立脑转移患者，包括大体积病灶，术后SRS/FSRT可以达到WBRT联合手术的局部控制效果，同时使58.4%-81%的患者免于接受WBRT^[35-38]^。

### 四 内科治疗

#### 1 NSCLC脑转移的化疗

化疗是NSCLC重要的综合治疗手段之一，也是NSCLC脑转移不可或缺的治疗手段。以顺铂、卡铂为主的铂类药物为基础，联合第三代细胞毒类药物可给NSCLC脑转移患者带来生存获益^[39-41]^。

培美曲塞在非鳞癌NSCLC中有良好的抗肿瘤活性，是非鳞癌NSCLC患者一线治疗和维持治疗的重要药物。培美曲塞联合铂类对NSCLC脑转移患者的颅内病灶也有控制作用，化疗组总生存（overall survival, OS）明显长于自然生存时间。GFPC07-01研究纳入初治的NSCLC脑转移患者，应用标准剂量的顺铂联合培美曲塞方案化疗6周期，化疗结束或者脑转移进展时进行WBRT治疗，脑转移病灶的有效率（overall response rate, ORR）为41.9%，颅外病灶的ORR为34.9%，中位OS为7.4个月^[40]^。培美曲塞可成为NSCLC脑转移患者一个有效的治疗选择^[41, 42]^。

替莫唑胺是一种新型的咪唑四嗪类烷化剂，可在人体内转化成有活性的烷化剂前体，能透过血脑屏障（blood-brain barrier, BBB），对于控制NSCLC脑转移有较好的疗效。对于既往接受过WBRT或全身化疗的NSCLC脑转移患者，可应用替莫唑胺以提高DCR、延长生存时间^[43]^。替莫唑胺（或联合其他化疗药物）与WBRT序贯或同步应用，尤其是同步应用，可提高颅内转移灶的DCR，为NSCLC脑转移患者提供新的治疗方法^[44, 45]^。目前相关报道多为Ⅱ期临床研究，显示替莫唑胺在NSCLC脑转移患者的治疗中安全、有效，但由于样本量较少，尚需大规模的Ⅲ期研究进一步证实。

#### 2 SCLC脑转移的化疗

化疗是SCLC脑转移患者综合治疗的一种有效手段。对于初治的SCLC脑转移患者，环磷酰胺/依托泊苷/长春新碱、顺铂/依托泊苷/长春新碱、环磷酰胺/阿霉素/依托泊苷三个化疗方案均具有一定疗效，脑转移病灶的ORR为27%-85%^[46-48]^。一线化疗对于脑转移病灶的疗效低于颅外病灶的疗效^[48]^。含铂的足叶乙甙或伊立替康二药方案是SCLC的标准一线化疗方案。卡铂单药治疗有症状的脑转移患者的ORR是44%，而卡铂联合伊立替康方案的疗效则是65%^[49]^。因此，建议对于广泛期SCLC伴有无症状的脑转移患者的一线治疗采用全身化疗，在全身化疗结束后或脑转移进展时再考虑WBRT。

已经有小样本研究显示，替尼泊苷和拓扑替康在SCLC脑转移治疗中具有一定的疗效和良好的安全性，可作为SCLC脑转移患者的治疗选择^[49-52]^。

#### 3 鞘内注射

鞘内注射化疗是将药物直接注入蛛网膜下腔，提高脑脊液内药物浓度，从而杀伤肿瘤细胞。给药途径包括：经腰椎穿刺蛛网膜下腔注射化疗药物和经Ommaya储液囊行脑室内化疗。与经腰椎穿刺鞘注给药相比，经Ommaya储液囊给药安全性更好，可避免鞘注误将药物注射到硬膜外间隙的风险；对于伴有血小板减少症的患者，可避免硬膜外和硬膜下血肿的发生。鞘内注射常用的化疗药物包括：甲氨蝶呤、阿糖胞苷和塞替派。鞘内注射化疗药物同时给予糖皮质激素可减轻化疗药物的神经毒性，缓解症状。腰椎穿刺时行脑脊液常规、生化及细胞学检查有助于监测疗效并指导治疗。鞘内化疗是NSCLC脑膜转移的重要治疗手段，对于脑实质转移，目前尚无明确支持证据。

#### 4 分子靶向治疗

尽管目前尚没有批准专门用于NSCLC脑转移的分子靶向治疗药物，但是近年来大量临床研究结果显示，分子靶向药物为NSCLC脑转移提供了新的治疗选择。

（1）EGFR-TKIs

越来越多学者关注EGFR-TKIs在NSCLC脑转移患者中的治疗作用。多数前瞻性Ⅱ期临床研究入组的患者为*EGFR*基因敏感突变高发的人群，如：东亚、非吸烟和腺癌等，ORR在32%-89%之间，中位无进展生存时间（progression-free surviral, PFS）在6.6个月-23.2个月，中位OS在12.9个月-21.9个月^[53-60]^。

EGFR-TKIs脂溶性好，能一定比例透过血脑屏障，对于NSCLC脑转移有治疗作用，可用于*EGFR*基因敏感突变的NSCLC脑转移患者的治疗^[61]^。对于*EGFR*基因敏感突变的NSCLC脑转移患者，EGFR-TKIs治疗可获得较好的客观缓解率。吉非替尼单药治疗*EGFR*基因敏感突变的肺腺癌伴脑转移患者的ORR为87.8%，中位颅内PFS为14.5个月，中位OS为21.9个月，吉非替尼治疗可显著延迟脑转移患者至放疗时间，中位至挽救性放疗时间为17.9个月，此外，EGFR 19号外显子缺失突变的患者较EGFR 21号外显子L858R突变的患者预后更好^[57]^。厄洛替尼二线治疗无症状的NSCLC脑转移的中位颅内PFS为10.13个月，中位OS为18.9个月^[58]^。BRAIN研究（CTONG1201）结果显示，与WBRT±化疗相比，埃克替尼显著改善了合并脑转移的*EGFR*基因敏感突变型晚期NSCLC患者的颅内PFS和颅内ORR，中位颅内PFS分别为4.8个月和10.0个月（HR=0.56, *P*=0.014）；6个月颅内PFS率分别为48.0%和72.0%（*P*＜0.001），颅内ORR分别为40.9%和67.1%（*P*＜0.001）^[62]^。

奥希替尼（Osimertinib, Tagrisso, AZD9291）为第三代EGFR-TKI，不可逆抑制*EGFR*基因敏感突变和T790M突变的肺癌细胞^[63]^。2015年11月13日美国食品药品监督管理局（Food and Drug Administration, FDA）批准奥希替尼上市，适应证为EGFR-TKI治疗进展后的*EGFR* T790M突变阳性的转移性NSCLC。动物实验显示，奥希替尼在脑组织中分布较吉非替尼和阿法替尼更高，药峰浓度（maximum concentration, Cmax）脑组织/血浆比（Brain/plasma Cmax ratio）在奥希替尼、吉非替尼和阿法替尼分别为3.41、0.21和＜0.36^[64]^。对于一线EGFR-TKI治疗后进展并伴有EGFR T790M突变的NSCLC脑转移患者，奥希替尼治疗与培美曲塞联合铂类化疗的PFS分别为8.5个月和4.2个月^[65]^。中国针对NSCLC脑转移的APOLLP研究（clinical trial号：NCT02972333）已经开始，以评估奥希替尼在中国EGFR-TKI治疗进展后的*EGFR* T790M突变阳性的脑转移NSCLC患者的疗效和安全性。

关于EGFR-TKIs联合WBRT或SRT是否可获益、毒性能否耐受，目前的前瞻性研究结论不甚一致，可能与入组人群选择与治疗方案不同有关，建议结合基因表达状态、组织学和临床数据（体能状态评分、胸部和其他颅外转移病灶情况和脑转移数目等）区分获益人群，并选择合适时机进行联合治疗^[66, 67]^。

在临床的医疗实践中，部分初治NSCLC脑转移患者服用EGFR-TKIs后原发病灶和脑转移灶同时得到缓解，对这样的患者还应择期适时进行SRT或WBRT。一般脑转移瘤体积越小的患者，采用SRS能获得更好的局部控制和对周围脑组织较小的损伤。

（2）ALK抑制剂

*ALK*融合基因是NSCLC另一个明确的治疗靶点。NSCLC患者*ALK*融合基因阳性率约为5%^[68]^。中国NSCLC患者*ALK*融合基因的阳性率约为3%-11%^[69, 70]^。克唑替尼是一种口服的ALK酪氨酸激酶受体抑制剂。与培美曲塞联合铂类化疗相比，克唑替尼对*ALK*融合基因阳性的NSCLC脑转移患者颅内转移瘤控制率更高^[71, 72]^。

对于克唑替尼治疗后进展的患者，可选择的新型ALK酪氨酸激酶受体抑制剂包括色瑞替尼（Ceritinib, LDK378）和阿雷替尼（Alecensa, Alectinib）等。Ⅱ期临床研究结果显示，阿雷替尼对于接受过克唑替尼治疗的*ALK*融合基因阳性的晚期NSCLC患者同样具有很好的疗效，尤其对于脑转移病灶，DCR为83%^[73]^。2015年12月11日美国FDA批准阿雷替尼上市，用于克唑替尼耐药的ALK阳性晚期NSCLC的治疗。

#### 5 抗血管生成药物

贝伐珠单抗是一种抗血管内皮生长因子（vascular endothelial growth factor, VEGF）的重组人源化单克隆抗体。贝伐珠单抗联合化疗对于非鳞NSCLC脑转移患者是安全、有效的^[74-76]^。回顾分析多项临床研究结果显示，无论是否应用贝伐珠单抗，脑转移患者出现脑出血的风险相似^[77]^。

### 五 对症治疗

肺癌脑转移患者常伴有颅内压升高导致的头痛、恶心、呕吐等症状，颅内高压的患者属于肿瘤急症，首先是积极给予脱水和利尿治疗以降低颅压，可选择的药物包括：甘露醇、甘油果糖和呋塞米。糖皮质激素，尤其是地塞米松可减轻脑水肿、改善脑转移患者的生活质量，但不改善预后。其次是控制症状，包括抗癫痫和镇痛治疗，由于抗癫痫药物不能减少NSCLC脑转移患者的癫痫发作次数，因此一般仅用于有发作症状的患者，不预防性应用^[78]^。头痛明显患者可予止痛对症治疗。

#### 1 甘露醇

20%甘露醇125 mL-250 mL静脉注射，依据症状每6-8小时一次，同时严密监测血浆电解质和尿量。甘露醇通过提高血浆渗透压，导致包括脑、脑脊液等组织内的水分进入血管内，从而减轻组织水肿，降低颅内压和脑脊液容量及其压力，可用于治疗脑转移瘤引起的脑水肿和颅高压，防止脑疝的发生。既往国内外动物及临床研究表明，甘露醇具有暂时性开放血脑屏障，促进肿瘤化疗药物向患者颅脑病灶渗透，提高颅内血药浓度及疾病缓解率的作用^[79]^。

#### 2 糖皮质激素

糖皮质激素是脑转移瘤周围水肿重要的治疗用药，具有改善肿瘤颅内转移相关症状的作用。其中地塞米松应用最为广泛，常与甘露醇联合使用。常用剂量是10 mg-20 mg静脉推注，然后4 mg-6 mg静脉注射，每6 h重复。高剂量（＞32 mg/d）有出现消化道出血等不良反应的风险，因此，大剂量应用一般不超过48 h-72 h。手术切除脑转移瘤前应用糖皮质激素可减轻术前及术后脑水肿，放疗时应用糖皮质激素可减轻早期放疗反应。需警惕糖皮质激素的副作用，防止消化性溃疡、血糖升高等不良反应的发生。糖尿病患者必须慎用糖皮质激素。

#### 3 利尿剂

呋塞米20 mg-40 mg静脉推注，依据颅内压增高程度、临床症状和24 h尿量调整剂量和频次，但须严密监测血浆电解质变化，尤其是低钠和低钾血症。

#### 4 抗癫痫治疗

部分肺癌脑转移患者在确诊前出现癫痫，亦有部分患者在病情发展过程中出现癫痫发作。应根据患者病情适时应用抗癫痫药物，并警惕抗癫痫治疗潜在的副作用，如肝功能异常、认知障碍和共济失调等。

## 六 预后

在分级预后系统（Graded Prognostic Assessment, GPA）基础上，根据不同原发肿瘤脑转移的差异进一步提出了诊断特异性GPA（diagnosis-specific, DS-GPA）。DS-GPA中，肺癌脑转移的预后因素包括年龄、卡氏评分（Karnofsky, KPS）、颅外转移和脑转移数目，具体评分标准如表 1。0分-1分、1.5分-2分、2.5分-3分和3.5分-4分NSCLC患者的中位OS分别为3.02个月、5.49个月、9.43个月和14.78个月；而0分-1分、1.5分-2分、2.5分-3分和3.5分-4分SCLC患者的中位OS分别为2.79个月、4.90个月、7.67个月和17.05个月。NSCLC和SCLC脑转移患者的中位OS分别为7.0个月和4.9个月^[80]^。

**1:** 肺癌脑转移分级预后系统预后评分标准

预后因素	0分	0.5分	1分
年龄（岁）	＞60	50-60	＜50
卡氏评分（分）	＜70	70-80	90-100
颅外转移	有	-	无
脑转移数目（个）	＞3	2-3	1

## 七 随访

肺癌脑转移患者诊治后应定期随访并进行相应的检查。检查方法包括病史、体格检查、血清肿瘤标志物检查、影像学检查等，频率一般为治疗后每2个月-3个月随访1次，病情变化时随时就诊，以根据病情变化采取相应的诊疗措施。

本专家共识的制订参考了国内外权威的肺癌诊疗指南以及最新的研究进展。由于临床实践中肺癌脑转移患者存在较大的个体差异，需根据具体情况决定每位患者的治疗策略，本共识仅供参考。

## 

专家组成员

**Table:** 

顾问	
孙燕	国家癌症中心/中国医学科学院北京协和医学院肿瘤医院内科，抗肿瘤分子靶向药物临床研究北京市重点实验室
于金明	山东省肿瘤医院放射治疗科
	
组长	
石远凯	国家癌症中心/中国医学科学院北京协和医学院肿瘤医院内科，抗肿瘤分子靶向药物临床研究北京市重点实验室
	
执笔人	
总执笔人	
石远凯	国家癌症中心/中国医学科学院北京协和医学院肿瘤医院内科，抗肿瘤分子靶向药物临床研究北京市重点实验室
影像诊断部分执笔人	
吴宁	国家癌症中心/中国医学科学院北京协和医学院肿瘤医院影像诊断科、PET-CT中心
分子病理部分执笔人	
王征	国家老年医学中心/北京医院病理科
血清肿瘤标志物部分执笔人
韩晓红	国家癌症中心/中国医学科学院北京协和医学院肿瘤医院检验科，抗肿瘤分子靶向药物临床研究北京市重点实验室
神经外科部分执笔人	
李学记	国家癌症中心/中国医学科学院北京协和医学院肿瘤医院神经外科
放疗部分执笔人	
肖建平	国家癌症中心/中国医学科学院北京协和医学院肿瘤医院放射治疗科
周宗玫	国家癌症中心/中国医学科学院北京协和医学院肿瘤医院放射治疗科

委员（排名不分先后，按姓氏笔画为序）	
丁翠敏	河北医科大学第四医院呼吸内科
马智勇	河南省肿瘤医院肿瘤内科
王子平	北京大学肿瘤医院胸部肿瘤内科
王东	第三军医大学大坪医院肿瘤中心
王征	国家老年医学中心/北京医院病理科
王孟昭	北京协和医院呼吸内科
王燕	国家癌症中心/中国医学科学院北京协和医学院肿瘤医院内科，抗肿瘤分子靶向药物临床研究北京市重点实验室
石远凯	国家癌症中心/中国医学科学院北京协和医学院肿瘤医院内科，抗肿瘤分子靶向药物临床研究北京市重点实验室
卢铀	四川大学华西医院肿瘤科
艾斌	国家老年医学中心/北京医院肿瘤内科
冯继锋	江苏省肿瘤医院肿瘤内科
刘云鹏	中国医科大学附属第一医院肿瘤内科
刘晓晴	中国人民解放军第307医院肿瘤科
刘基巍	大连医科大学附属第一医院肿瘤科
伍钢	华中科技大学协和医院肿瘤中心
曲宝林	中国人民解放军总医院放射治疗科
李学记	国家癌症中心/中国医学科学院北京协和医学院肿瘤医院神经外科
李恩孝	西安交通大学第一附属医院肿瘤内科
李薇	吉林大学第一医院肿瘤科
宋勇	中国人民解放军南京总医院呼吸内科
陈公琰	哈尔滨医科大学附属肿瘤医院肿瘤内科
陈正堂	第三军医大学新桥医院肿瘤科
陈骏	大连医科大学附属第二医院肿瘤内科
余萍	四川省肿瘤医院肿瘤内科
吴宁	国家癌症中心/中国医学科学院北京协和医学院肿瘤医院影像诊断科、PET-CT中心
吴密璐	青海大学附属肿瘤医院肿瘤内科
肖文华	中国人民解放军总医院第一附属医院肿瘤内科
肖建平	国家癌症中心/中国医学科学院北京协和医学院肿瘤医院放射治疗科
张力	北京协和医院呼吸内科
张阳	大连医科大学附属第二医院肿瘤内科
张沂平	浙江省肿瘤医院肿瘤内科
张树才	首都医科大学附属北京胸科医院肿瘤内科
宋霞	山西省肿瘤医院呼吸内科
罗荣城	南方医科大学肿瘤中心肿瘤内科
周彩存	同济大学附属上海市肺科医院肿瘤科
周宗玫	国家癌症中心/中国医学科学院北京协和医学院肿瘤医院放射治疗科
赵琼	浙江大学附属第一医院胸部肿瘤科
胡成平	中南大学湘雅医院呼吸内科
胡毅	中国人民解放军总医院肿瘤内科
聂立功	北京大学第一医院呼吸内科
郭其森	山东省肿瘤医院肿瘤内科
常建华	复旦大学附属肿瘤医院肿瘤内科
黄诚	福建省肿瘤医院肿瘤内科
韩宝惠	上海交通大学附属胸科医院呼吸内科
韩晓红	国家癌症中心/中国医学科学院北京协和医学院肿瘤医院检验科，抗肿瘤分子靶向药物临床研究北京市重点实验室
黎功	中国武警总医院肿瘤科
	
学术秘书	
黄昱	国家癌症中心/中国医学科学院北京协和医学院肿瘤医院内科，抗肿瘤分子靶向药物临床研究北京市重点实验室
史幼梧	国家癌症中心/中国医学科学院北京协和医学院肿瘤医院内科，抗肿瘤分子靶向药物临床研究北京市重点实验室
